# Interplay between Peptide Bond Geometrical Parameters in Nonglobular Structural Contexts

**DOI:** 10.1155/2013/326914

**Published:** 2013-12-26

**Authors:** Luciana Esposito, Nicole Balasco, Alfonso De Simone, Rita Berisio, Luigi Vitagliano

**Affiliations:** ^1^Institute of Biostructures and Bioimaging, CNR, Via Mezzocannone 16, 80134 Napoli, Italy; ^2^Seconda Università di Napoli, Via Vivaldi 43, 81100 Caserta, Italy; ^3^Division of Molecular Biosciences, Imperial College South Kensington Campus, London SW7 2AZ, UK

## Abstract

Several investigations performed in the last two decades have unveiled that geometrical parameters of protein backbone show a remarkable variability. Although these studies have provided interesting insights into one of the basic aspects of protein structure, they have been conducted on globular and water-soluble proteins. We report here a detailed analysis of backbone geometrical parameters in nonglobular proteins/peptides. We considered membrane proteins and two distinct fibrous systems (amyloid-forming and collagen-like peptides). Present data show that in these systems the local conformation plays a major role in dictating the amplitude of the bond angle N-C^*α*^-C and the propensity of the peptide bond to adopt planar/nonplanar states. Since the trends detected here are in line with the concept of the mutual influence of local geometry and conformation previously established for globular and water-soluble proteins, our analysis demonstrates that the interplay of backbone geometrical parameters is an intrinsic and general property of protein/peptide structures that is preserved also in nonglobular contexts. For amyloid-forming peptides significant distortions of the N-C^*α*^-C bond angle, indicative of sterical hidden strain, may occur in correspondence with side chain interdigitation. The correlation between the dihedral angles Δ*ω*/*ψ* in collagen-like models may have interesting implications for triple helix stability.

## 1. Introduction

Protein three-dimensional structures are characterized by a high level of complexity [1]. Intriguingly, this complexity is coupled with marginal thermodynamic stabilities. Indeed, these intricate structures are the result of the delicate balance of a variety of different contributions. Even minor changes may undermine the overall organization of these macromolecules. In this framework, a comprehensive description of protein structures should not neglect subtle details that may become fundamental in specific situations.

In the last two decades there has been considerable interest in the analysis of protein backbone geometry. Several independent investigations have provided a plastic view of the protein backbone geometry [2–18]. These statistical and quantum chemical investigations have highlighted an interesting interplay between them. In particular, it has been shown that many backbone bond angles strongly depend on the local conformations [2, 10]. Similarly, the local conformation also affects peptide bond distortions from planarity and the pyramidalization of the carbonyl carbon atom [5, 19]. Finally, a correlation between bond distances such as CO and CN has been detected in ultrahigh resolution protein structures [4]. It is important to note, however, that correlations between backbone geometrical parameters have been disclosed by performing analyses on protein structure databases essentially made of globular proteins. Since protein structures are extremely sensitive to the local context, we here evaluated the occurrence of such correlations in nonglobular systems. In particular, we considered the transmembrane regions of membrane proteins and two different fibrous models: the collagen triple helix [20–22] and the steric-zipper motif exhibited by amyloid-like peptides [23, 24]. These systems were selected to evaluate the impact on the protein geometry variability exerted by (a) the polarity of the medium in which proteins are immersed and (b) the structural strain present in fibrous proteins. The feasibility of the present study has been facilitated by the recent progresses of the crystallographic techniques, which have increased the number and the accuracy of structures of these nonglobular systems. It is worth mentioning that, although these proteins are underrepresented in current structure databases, they are very frequent in nature. Indeed, collagen is the most abundant protein in vertebrates [25]. Moreover, it has been estimated that membrane proteins may represent nearly 40% of the total protein content in the human genome [26]. Finally, recent data show that the tendency to form amyloid-like fibers is a rather common property of proteins [27].

## 2. Methods

### 2.1. Ensembles and Definition

Statistical surveys of peptide bond geometrical parameters were performed on specific classes of protein/peptide structures reported in the Protein Data Bank (PDB) (release of March 2013) [28]. As detailed below, for each class different selection criteria were applied.

**Figure 1:**
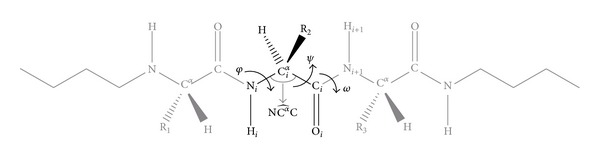
Schematic representation showing the backbone torsion angles *φ*, *ψ*, and  *ω* and the bond angle N–C^*α*^–C. *ω*_3_ is the dihedral angle defined by the atoms O_*i*_C_*i*_N_*i*__+1_C_*i*+1_^*α*^.

Peptide bond distortions were evaluated by using the Δ*ω* defined as (*ω* − 180°) mod360°. Carbon carbonyl pyramidalization *ϑ*_C_ was measured as (*ω* − *ω*_3_ + 180°) mod360°, where *ω*_3_ is the dihedral angle defined by the atoms O_*i*_C_*i*_N_*i*+1_C_*i*+1_^*α*^ (Figure 1). 

*(a)Membrane Proteins.* Structures were selected considering the nonredundant ensemble (sequence identity lower than 40%) of proteins containing transmembrane regions by using the website PDBTM [29]. Only structures refined at a resolution better than 2.0 Å were considered. This selection yielded the following 47 protein chains isolated from 32 PDB structures (the last letter in the code is the chain identifier): 3m71a; 3b9wa; 2w1pa; 2a65a; 1kqfc; 1kqfb; 2bs2c; 2b2fa; 1yc9a; 3egwc; 3ddla; 3cx5d; 2pora; 3fida; 2o4va; 2gufa; 1xioa; 1kmoa; 2wjqa; 2erva; 1xkwa; 3abkm; 3abkl; 3abkk; 3abkj; 3abki; 3abkg; 3abkd; 3abkb; 3a7ka; 3qraa; 2y69d; 2y69g; 2y69i; 2y69j; 2y69k; 2y69l; 2y69m; 3arci; 3arct; 3gwoa; 3nyma; 4dx5; 4eiya; 2f93a; 2x7ra; 3rlba. The resolution of these structures is reported in Figure S1A in Supplementary Material available online at http://dx.doi.org/10.1155/2013/326914.

By using the annotation of PDBTM, we selected 4494 transmembrane residues from these structures. These residues were used in the analysis of the Δ*ω*/*ψ* correlations. Gly and Pro residues, which present nonglobular geometrical parameters, were excluded from the analysis of the N–C^*α*^–C angle (also referred to as *τ*) dependency on the conformation. The assignment of secondary structure was carried out by using the program DSSP [30].

*(b) Amyloid-Like Peptides.* The following twenty-eight steric-zipper structures present in the PDB were identified by searching manually the database using the literature information: 1yjo, 1yjp, 2okz, 2ol9, 2olx, 2on9, 2onv, 2onw, 2onx, 3dg1, 3dgj, 3fod, 3fpo, 3fqp, 3ftk, 3ftl, 3ftr, 3fva, 3hyd, 3nhc, 3loz, 3nvh, 3nvg, 3nvf, 3nve, 3ow9, 3q2x, and 3pzz. Only structures refined at a resolution better than 1.8 Å were considered. A single copy of each peptide was considered when multiple copies were present in the asymmetric unit of the crystal. The distribution of the resolution of these structures is reported in Figure S1B. From these structures 114 residues were selected by omitting terminal aminoacids that are generally charged in these peptides.

*(c) Collagen-Like Polypeptides*. Geometrical correlations in triple helix models were analyzed by considering only highly accurate structures of collagen-like polypeptides. It is well known that crystals of these polypeptides are frequently disordered. Therefore, we selected a subset of collagen-like models whose structure was determined at atomic resolution. In particular, we considered the structure of the peptides (Pro-Pro-Gly)_9_ (PDB code 3ah9, resolution 1.08 Å, *R*_factor_ 0.165, and *R*_free_ 0.191), (Pro-Pro-Gly)_4_-Hyp-Asp-Gly-(Pro-Pro-Gly)_4_ (PDB code 3abn, resolution 1.02 Å, *R*_factor_ 0.131, and *R*_free_ 0.163) [31], (Pro-Pro-Gly)_4_-Hyp-Val-Gly-(Pro-Pro-Gly)_4_ (PDB code 3a0 m, resolution 1.02 Å, *R*_factor_ 0.116, and *R*_free_ 0.143), and (Pro-Pro-Gly)_4_-Hyp-Thr-Gly-(Pro-Pro-Gly)_4_ (PDB code 3a1 h, resolution 1.08 Å, *R*_factor_ 0.135, and *R*_free_ 0.185) [32].

## 3. Results and Discussion

To evaluate the occurrence of correlations between peptide geometry and conformation in nonglobular contexts we here analyzed structures of membrane and fibrous (amyloid-like and collagen) peptides/proteins. Although, in principle, *ω*, *ϑ*_C_, and N–C^*α*^–C depend on both *φ* and *ψ*, the analysis of the literature trends clearly shows a crucial role of the *ψ* angle in modulating these parameters [2, 6, 7]. Therefore, all subsequent analyses were carried as a function of *ψ*.

*(a) Membrane Proteins*. The detection of correlations between peptide geometry and conformation typically requires highly accurate protein structures that are refined against ultrahigh resolution diffraction data. However, the conformational dependency of some geometrical parameters can also be detected in structures determined at medium-high resolution (~2.0 Å). This holds for (a) the N–C^*α*^–C bond angle and for (b) the Δ*ω*/*ψ* correlation [2, 5]. This observation offers the possibility to check the occurrence of these correlations also in proteins whose structures were generally determined at moderate resolution such as membrane proteins.

**Figure 2:**
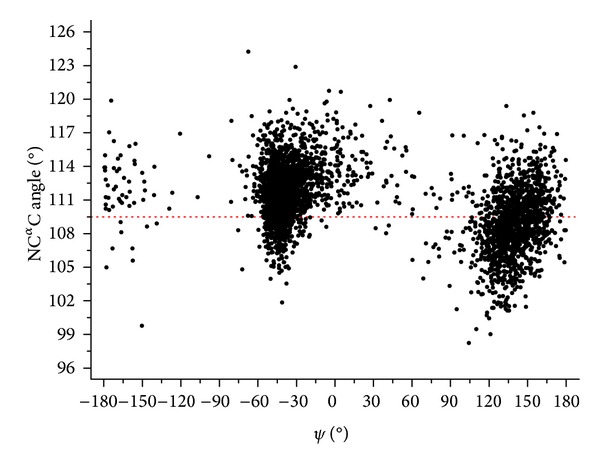
(N–C^*α*^–C) versus *ψ* for the transmembrane residues of the membrane protein ensemble. The dotted red line represents the ideal value for an ideal tetrahedral geometry (109.5°).

As reported in detail in the Methods section, we conducted these analyses on a nonredundant set of membrane proteins, sharing sequence identities lower than 40%. Only the transmembrane segments of these proteins were selected. As expected, most of the residues of these regions adopt either *α*-helical (2415 residues) or *β*-sheet (1133 residues) conformations (Figure S2). A minor fraction of residues (75 residues) adopting the structure of a 3–10 helix was also observed. As shown in Figure 2, there is a clear dependency of N–C^*α*^–C on the *ψ* angle. In particular, the N–C^*α*^–C angle assumes the largest values in 3–10 helices (112.8° ± 2.2°) and the smallest ones in *β*-sheets (108.6° ± 2.9°) (Figure S3). Intermediate values are detected for *α*-helices (111.3° ± 2.0°) (Figure S3). The significance of these differences has been evaluated by using the two-sided two-sample Student's *t*-test. These analyses indicate that the differences between mean values of the pairs *α*-helix/3–10 helix, *α*-helix/*β*-sheet, and 3–10 helix/*β*-sheet are statistically significant at *P* = 0.001. Previous studies carried out on globular proteins have shown that *τ* assumes the largest values when *ψ* approaches to 0° [2, 6]. A second minor maximum is assumed for *ψ* values 150°–180° whereas minimal *τ* values are observed for *ψ* ~ 90°. Our data show that the average values of the regions characterized by −5° < *ψ* < 5°, 160° < *ψ* < 170°, and 85° < *ψ* < 95° are 114.2° ± 2.6°, 110.5° ± 2.4°, and 109.0° ± 4.3°, respectively. Therefore, the N–C^*α*^–C variability observed in membrane proteins perfectly fits into the scheme derived for globular proteins. This suggests that the local polar/apolar environment does not play any role in modulating this geometrical parameter.

**Figure 3:**
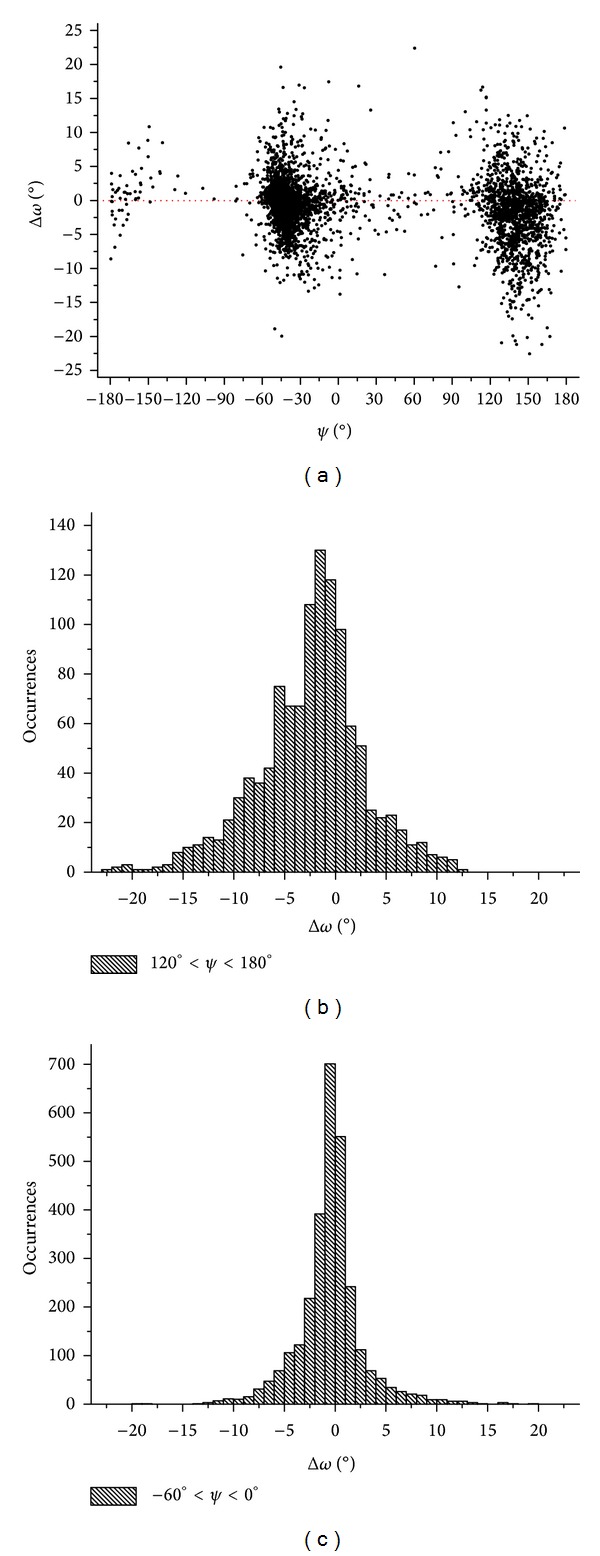
Δ*ω* variability in transmembrane residues. In panel (a), Δ*ω* versus *ψ* is reported. The distribution of Δ*ω* values exhibited by residues of *α*-helices and *β*-sheets is reported in panels (b) and (c), respectively.

The analysis of the *ψ*/*ω* values indicates the occurrence of this correlation in membrane proteins (Figure 3(a)). In particular, residues assuming a *β*-conformation, whose *ψ* falls in the interval 120–180°, show a tendency to adopt negative Δ*ω* values (average Δ*ω* value −2.0°) (Figure 3(b)). This is in line with data observed for the general ensemble of protein structures which indicate that residues with *ψ* values in the interval 120°–180° display negative Δ*ω* values [5, 7, 11].

The analysis of the Δ*ω* distribution in *α*-helical residues does not display, on average, any significant deviation from planarity (Figure 3(c)). This is also in line with previous analysis conducted on globular proteins which showed that helical residues display minimal deviations, with both positive and negative Δ*ω* values, from planarity [5, 7, 11].

Altogether, these findings suggest that a large variation of the local polarity, going from soluble globular proteins to membrane proteins, does not affect peptide bond distortion from planarity. They also imply that the electronic effects that dictate peptide bond deformations [7] also operate in apolar contexts.

We also checked the occurrences of specific trends of carbonyl C-pyramidalization in these membrane protein structures. These analyses do not highlight any clear trend (data not shown). This observation may be ascribed to the limited resolution of the structures available.

**Figure 4:**
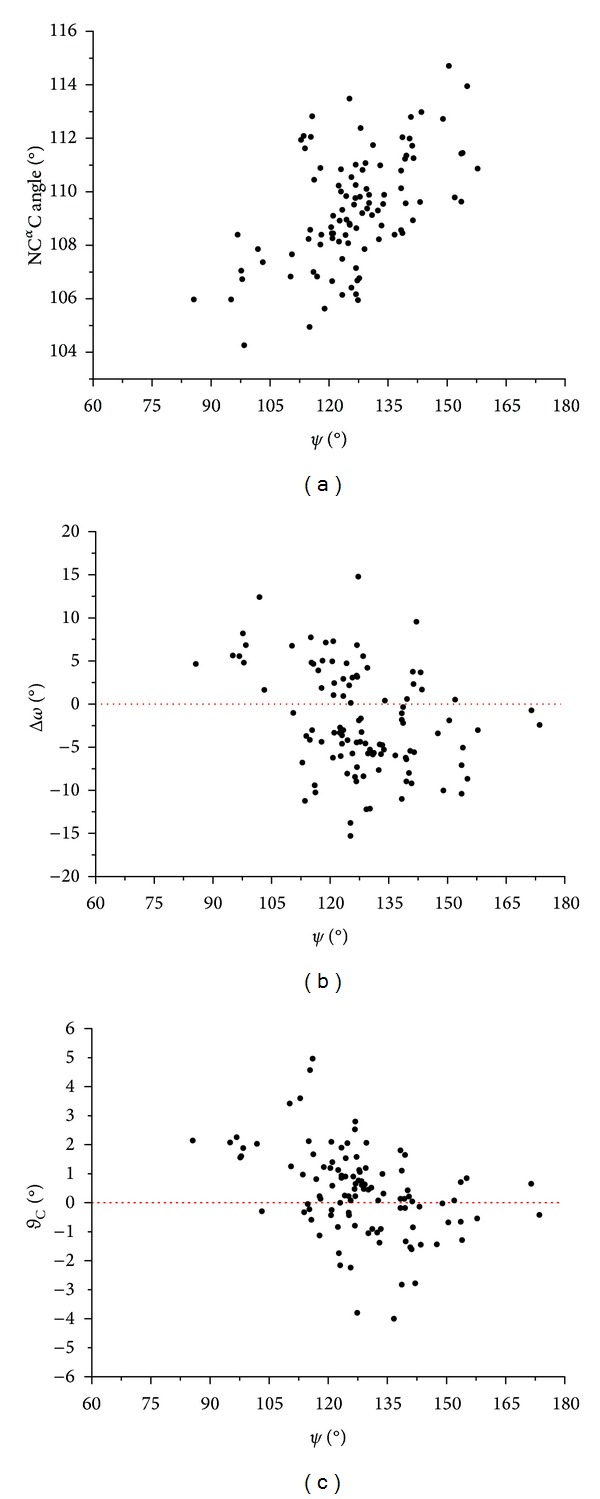
Dependence of (N–C^*α*^–C) (a), Δ*ω* (b), and *ϑ*_C_ (c) as function of *ψ* for residues of peptides forming amyloid-like fibers.

**Figure 5:**
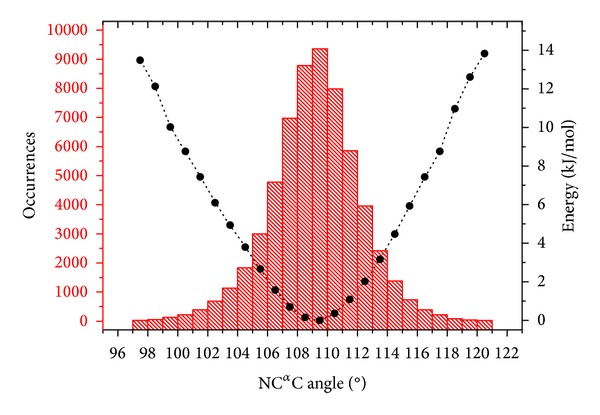
The histogram represents the distribution of (N–C^*α*^–C) angles for residues located in *β*-sheets derived from 5139 protein chains extracted from the PDB. These chains were selected by applying the following criteria: resolution better than 2.2 Å, *R*_factor_ lower than 0.20, and mutual sequence identities lower than 25%. The black points represent energies derived from Maxwell-Boltzmann relationship *n*_*i*_ = *n*_*o*_ exp(−Δ*E*/*kT*), where *n*_*i*_ is the number of observations in state *i*, *n*_*o*_ is the number of observations in the most populated state, *k* is the Boltzmann factor, *T* is the temperature of the system, and Δ*E* is the energy difference between two energy states.

*(b) Steric-Zipper Motif in Amyloid-Like Peptides*. It has been recently discovered that amyloidogenic fibril-forming peptides assume a rather unusual structure characterized by the tight association of the side chains belonging to two-facing *β*-sheets (steric-zipper motif) [23, 24]. It is believed that this motif represents a reliable surrogate of amyloid-like fibrils formed by protein and peptides associated with the insurgence of neurodegenerative diseases. It has been proposed that the tight interdigitation of side chains in the steric-zipper models confers an elevated structural stability to this motif. We evaluated here the impact of this tight interdigitation on the geometrical parameters of 28 high resolution steric zipper structures, in relation to their dependence on the conformation. The average value of the N–C^*α*^–C angle is 109.4°. This value is only slightly higher than that observed for residues in *β*-structure of globular proteins (109.2°). Interestingly, the evaluation of the *τ*/*ψ* correlation clearly indicates that *τ* riseswhen *ψ* increases from 90 to 180° (correlation coefficient of 0.55 with a *P* value <10^−4^) (Figure 4(a)). This is the trend typically observed in globular proteins. It is worth mentioning, however, that some of the larger deformations of the N–C^*α*^–C angle (>112°) observed in these fibrillar structures occur when the interdigitation of residues between facing sheets is stronger. In Figure S4, some examples are reported in which the tight interdigitation leads to a significant deformation of the N–C^*α*^–C angle. A rough estimation of the energetic cost associated with the deformation of this angle was obtained by plotting the distribution of its values and by deriving energies according to the Maxwell-Boltzmann relationship (Figure 5). The analysis of the resulting diagram indicates that the energy cost for a deformation of N–C^*α*^–C to 114°, a value that may be observed in concomitance with tight interdigitation (Figure S4), is about 4 kJ per mol at the temperature of 298 K (Figure 5). Although the absolute value of this energetic term is limited, it may be important in repetitive systems as amyloid-like fibrils. Therefore, the cost associated with the backbone geometry deformation is one of the factors that should be considered in the energetic balance of the process that leads to amyloid-like fiber formation. It is worth mentioning that some *β*-branched residues (Ile and Val) have a lower propensity to assume large N–C^*α*^–C values [18]. This implies that, for these residues, a larger energetic cost is associated with the deformations required for these tight associations. Indeed, the energetic cost, estimated by using the Maxwell-Boltzmann approach described above, for the Ile residue to adopt a N–C^*α*^–C angle of 114° is about 5 kJ per mol at 298 K (data not shown). These considerations suggest that specific residue substitutions in key points of amyloid-like protein/peptides may favor or disfavor the process of fibril formation. Although this analysis represents one of the first quantifications of the energetics involved in the geometry distortion caused by steric zipper, future systematic studies by using quantum chemistry approaches are likely to provide further interesting insights into this topic.

Present data indicate that the *ψ*/*ω* correlation is also observed. Indeed, as shown in Figure 4(b), Δ*ω* values are positive and negative when *ψ* approaches values close to 90° and 150°, respectively. Since these peptides present elevated resolutions and good crystallographic factors, we also evaluated the dependence of the *ϑ*_C_ pyramidalization on *ψ*. As shown in Figure 4(c), there is a clear dependence of *ϑ*_C_ on *ψ*, which strictly follows the one observed in high resolution structures of globular proteins. Altogether, these findings demonstrate that the interplay of different geometrical parameters is preserved even in these highly strained systems.

*(c) Collagen-Like Polypeptides*. Collagen is characterized by an uncommon abundance of glycine and iminoacid residues that are regularly distributed in the sequence in repetitive triplets of the type Gly-X-Y [20, 21, 25, 33, 34]. Although the three residues of this motif assume a polyproline PPII conformation, they present distinct (*φ*, *ψ*, *ω*) values. Indeed, structural studies carried on model peptides of the type Pro-Pro-Gly or Pro-Hyp-Gly have shown that *ψ* adopts values close to 175°, 165°, and 150° for Gly, Pro in *X*, and Pro in *Y* (or Hyp), respectively. Although crystals of collagen-like peptides frequently show significant disorder and/or twinning, very recently ultrahigh resolution (better than 1.1 Å) models have been reported. Correlations among geometrical parameters were initially analyzed here by considering the structure of (Pro-Pro-Gly)_9_. Given the peculiar aminoacid composition of these peptides, the distribution of N–C^*α*^–C values was not considered; the analyses were, therefore, restricted to Δ*ω*/*ψ* and *ϑ*_C_/*ψ* correlations.

**Figure 6:**
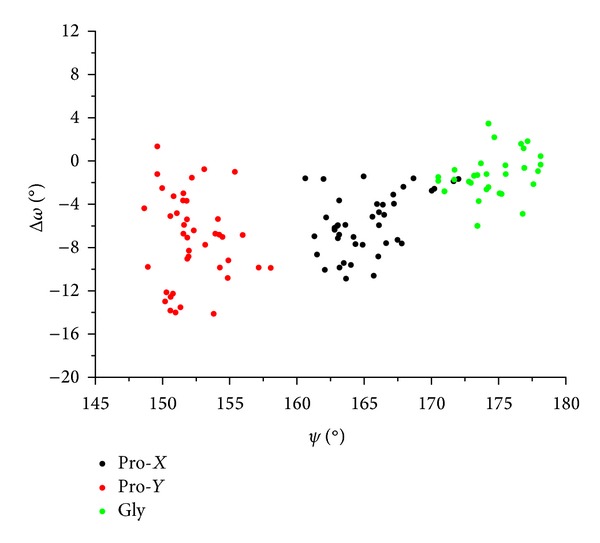
Δ*ω* versus *ψ* for residues of the collagen-like peptide (Pro-Pro-Gly)_9_.

As shown in Figure 6, the values of Δ*ω* clearly depend on *ψ*. In particular, Gly residues, which display a *ψ* angle close to 180°, present a virtually planar peptide bond (Δ*ω* = −1.3° ± 2.2). Significant distortions from planarity are displayed by both Pro residues in position *X* (Δ*ω* = −5.8° ± 2.8) and in position *Y* (Δ*ω* = − 7.2° ± 4.1). Notably, Pro residues in *Y*, which show *ψ* angles close to 150°, display larger deviations from planarity than Pro residues in *X*. Similar results are obtained from the analyses carried out on Gly, Pro-*X*, and Pro-*Y* residues isolated from other ultrahigh resolution structures of collagen-like peptides (data not shown). The evaluation of the dependence of *ϑ*_C_ pyramidalization on *ψ* leads to less clear results. Although, as expected, *ϑ*_C_ presents average negative values for these residues, the entity of its absolute value (~−1°) is likely not significant (Figure S5).

The trends detected here are in line with the Δ*ω*/*ψ* dependence reported for globular proteins. Not only do these findings assess the high accuracy of these collagen-like structures, but they also hold important implications for the triple helix buildup. Indeed, previous studies have shown that the interplay between dihedral angles played a major role in the stabilization/destabilization of the triple helix [20, 21]. In particular, limited variations of the main-chain dihedral angles associated with different proline puckering have dramatic effects on triple helix stability. The present findings further extend the notion of dihedral angle correlations in the triple helix to Δ*ω*/*ψ*. By influencing *φ* and *ψ*, proline puckering also dictates the propensity of the peptide bond to undergo distortions. It is important to note that the CO group of the Pro in *X* is involved in the H-bond interactions with the N nitrogen atom of the Gly residues, which stabilize the triple helix. In this scenario, it can be suggested that the positional preference for the down pucker of Pro in *X* is related to the interplay of main-chain dihedral angles that leads to the optimal position of the CO for the H-bond formation. Although the magnitude of the peptide bond deviations is limited, its significance is increased in a repetitive system as collagen triple helix. These considerations support the concept that the stability of protein structures may rely on subtle effects, which can be highlighted only by accurate protein structure determinations.

## 4. Conclusions

Present data clearly indicate that the interplay of backbone geometrical parameters is an intrinsic and general property of protein/peptide structure that is preserved also in highly nonglobular contexts. The detection of these features in protein structures is therefore exclusively related to their accuracy. In line with previous suggestions [7, 11], these correlations should be systematically checked in protein structure validation protocols. Since the subtle correlations of the geometrical parameters become more evident with the increase of resolution, their evaluation may represent a tool particularly indicated for the validation of high/ultrahigh resolution protein structures. The general validity of these subtle observed trends also prompts their use in protein refinement protocols [35] as well as in the development of accurate force fields [2] for computational studies.

## Supplementary Material

Fig. S1 reports the distribution of the resolution of membrane protein and amyloid-like peptide crystal structures considered in the present study.
Fig. S2 reports the overall Ramachandran plot for residues belonging to the transmembrane regions of membrane proteins.
Fig. S3 reports the distribution of N-C^*α*^-C values for residues in *α*-helices, *β*-sheets and 3-10 helices in the selected dataset of membrane protein structures.
Fig. S4 reports some examples in which the tight interdigitation leads to a significant deformation of the N-C^*α*^-C angle in amyloid-like peptides.
Fig S5 reports the dependence of *θ*_C_ pyramidalization on *ψ* for residues of the collagen-like peptide (Pro-Pro-Gly)_9_.

## Abbreviations

PDB: Protein Data Bank
*τ*: N–C^*α*^–C bond angle
Hyp: Three-letter code for (2S,4R)-hydroxyproline.
