# Supplementary material for: Interplay between Peptide Bond Geometrical Parameters in Nonglobular Structural Contexts
Source: Biomed Res Int. 2013 Dec 26;2013:326914. doi: 10.1155/2013/326914 (PMC3888713; doi:10.1155/2013/326914)
Supplement: Supplementary file 1 — Fig. S1 reports the distribution of the resolution of membrane protein and amyloid-like peptide crystal structures considered in the present study. Fig. S2 reports the overall Ramachandran plot for residues belonging to the transmembrane regions of membrane proteins. Fig. S3 reports the distribution of N-Cα-C values for residues in α-helices, β-sheets and 3-10 helices in the selected dataset of membrane protein structures. Fig. S4 reports some examples in which the tight interdigitation leads to a significant deformation of the N-Cα-C angle in amyloid-like peptides. Fig S5 reports the dependence of θ C pyramidalization on ψ for residues of the collagen-like peptide (Pro-Pro-Gly)9. [file 326914.f1.pdf]

Supplementary Material

Interplay between peptide bond geometrical parameters in non-globular structural contexts

Luciana Esposito, Nicole Balasco, Alfonso De Simone, Rita Berisio, Luigi Vitagliano.

A

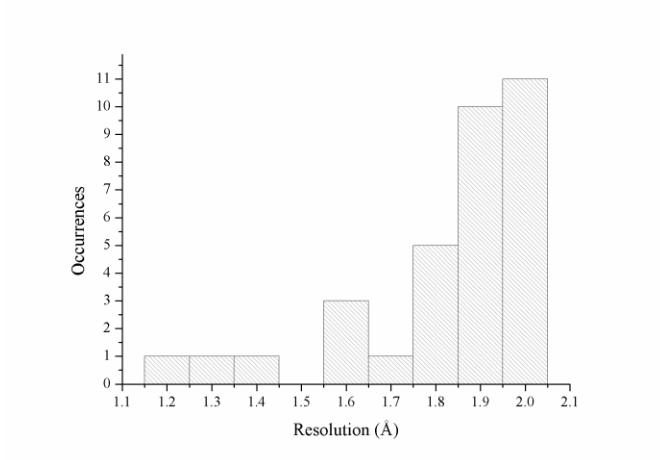

B

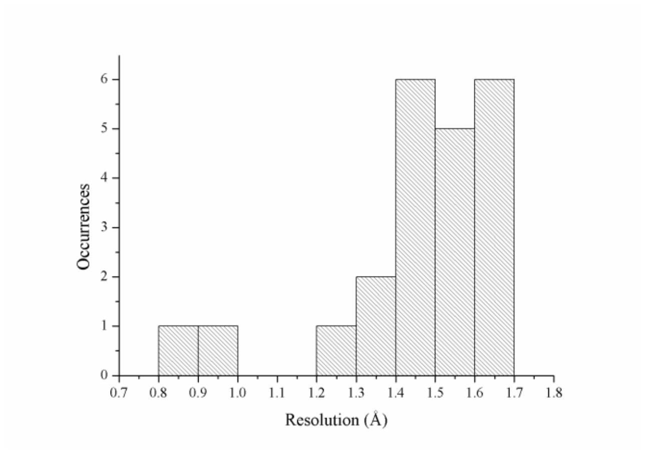

Figure S1: Distribution of the resolution of membrane protein (A) and amyloid-like peptide (B) crystal structures considered in the present study.

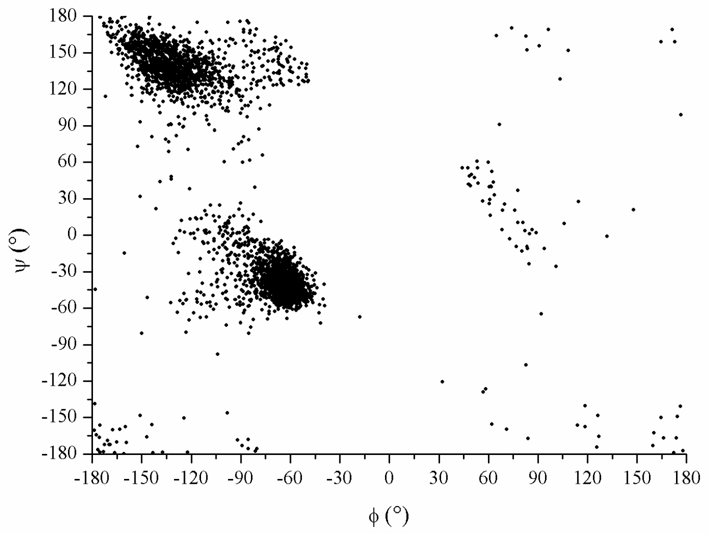

Figure S2: Overall Ramachandran plot for residues of membrane protein structures.

A

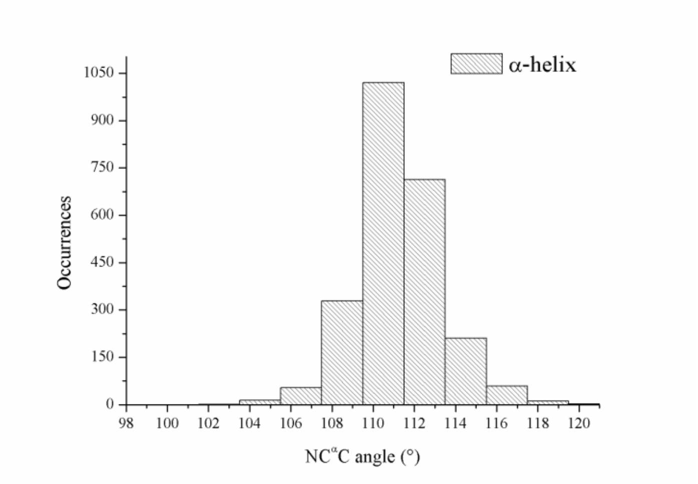

B

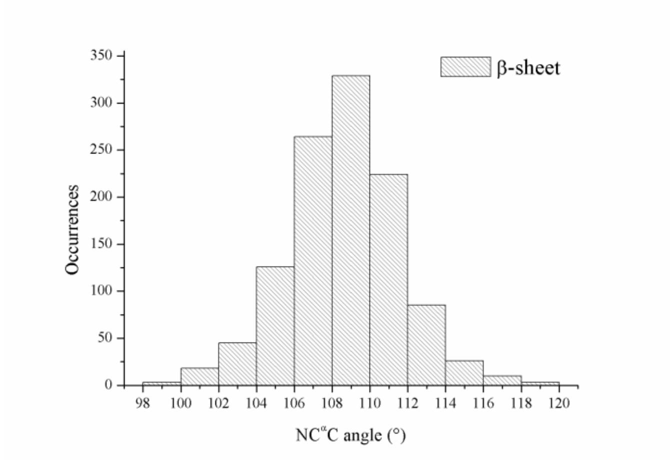

C

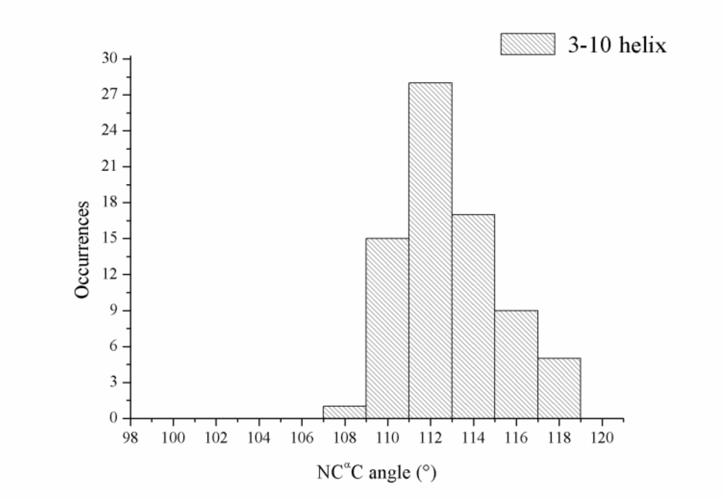

Figure S3. Distribution of N-C$^{\alpha}$-C values for residues in α-helices (A), β-sheets (B) and 3-10 helices (C) of membrane proteins.

A

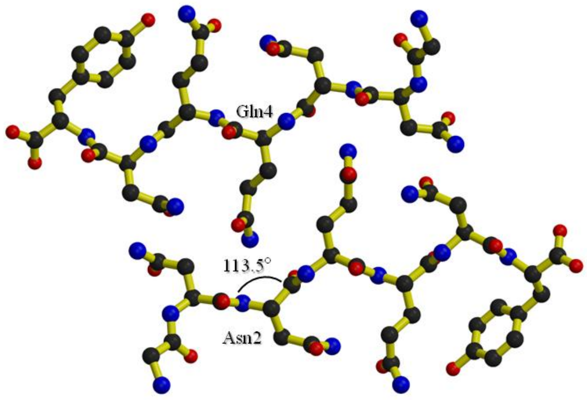

B

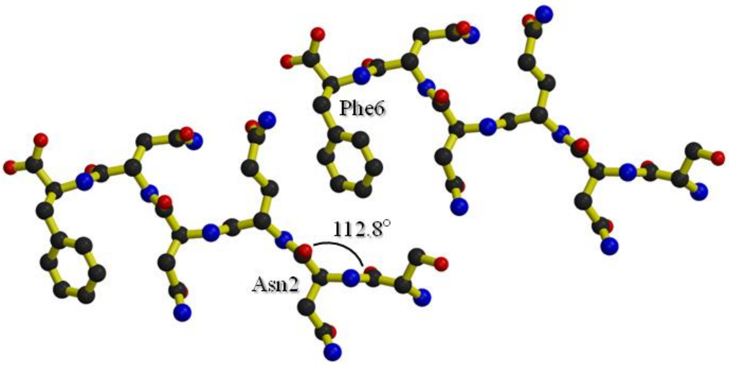

C

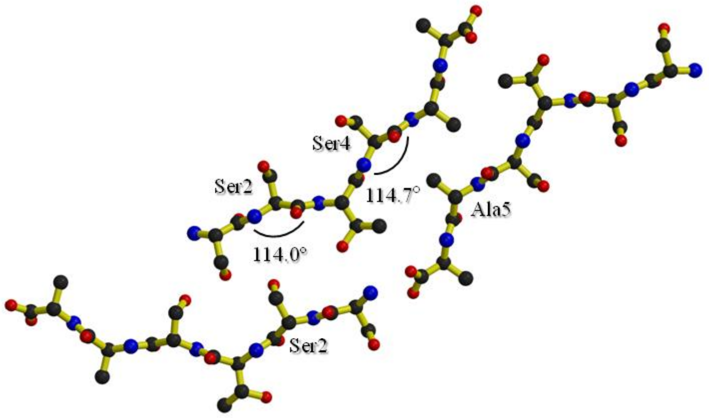

Figure S4. Examples of steric zipper assemblies: GNNQQNY (A), SNQQNF (B), SSTSSA (C). The deformation of some angles induced by interdigitation is shown.

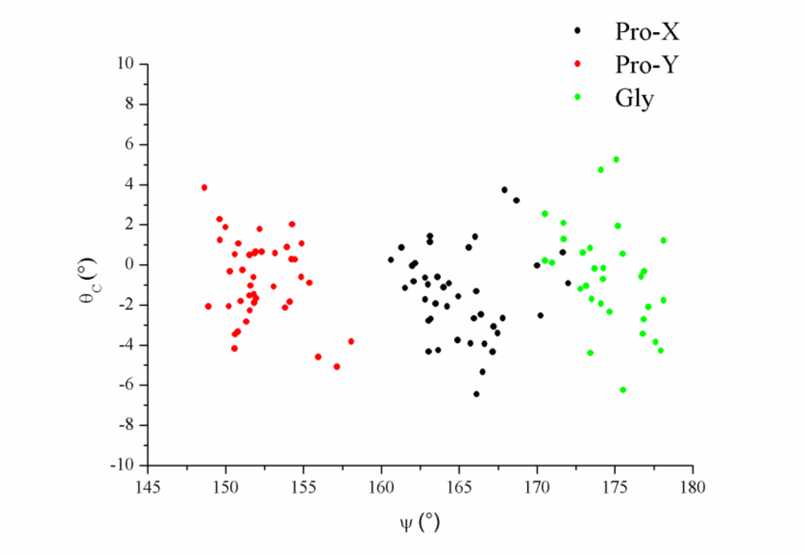

Figure S5: ϑ_C *versus* ψ for residues of the collagen-like peptide (Pro-Pro-Gly)$_9$.
